# Prof. C. B. Coventry

**Published:** 1850-01

**Authors:** 


*Prof. C. B. Coventry.*—The course of instruction by Prof. Coventry on Physiology and Medical jurisprudence, in the University of Buffalo, will commence with the commencement of the present month.

